# Tracheobronchial amyloidosis: an uncommon disease with a common presentation

**DOI:** 10.1002/rcr2.630

**Published:** 2020-08-02

**Authors:** Loveleen Mangla, Rohit Vadala, Shirish Kumar Kadli, Deepak Prajapat, Deepak Talwar

**Affiliations:** ^1^ Metro Centre for Respiratory Diseases (MCRD) Metro Multispeciality Hospital Noida India

**Keywords:** Amyloid, haemoptysis, heterogeneous, local disease, tracheobronchial amyloidosis

## Abstract

Amyloidosis is an uncommon heterogeneous and multi‐systemic disease characterized by extracellular amyloid deposition. The size of proteins varies and forms a part of local disease or systemic process. Light chain amyloidosis (AL) is the most prevalent form of systemic amyloidosis which may also be seen in localized disease. Isolated tracheobronchial amyloidosis (TBA) is rather unusual with local amyloid deposition which may pose a diagnostic dilemma with subsequent therapeutic challenge. Awareness of such a presentation is crucial in the diagnosis of this rare disease. We describe three cases who presented with haemoptysis, which on further evaluation were diagnosed as isolated TBA, and a review of literature.

## Graphical abstract

We describe three cases who presented with haemoptysis, which on further evaluation were diagnosed as isolated tracheobronchial amyloidosis, and a review of literature.

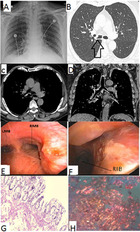

## Introduction

Amyloidosis is an uncommon heterogeneous and multi‐systemic disease characterized by extracellular amyloid deposition. Isolated tracheobronchial amyloidosis (TBA) is rather unusual with local amyloid deposition which may pose as a diagnostic dilemma with subsequent therapeutic challenge. Awareness of such a presentation is crucial in the diagnosis of this rare disease. We describe three cases who presented to our facility with haemoptysis, which on further evaluation were diagnosed as isolated TBA, and a review of literature.

## Case Series

### Case 1

**Figure 1:**
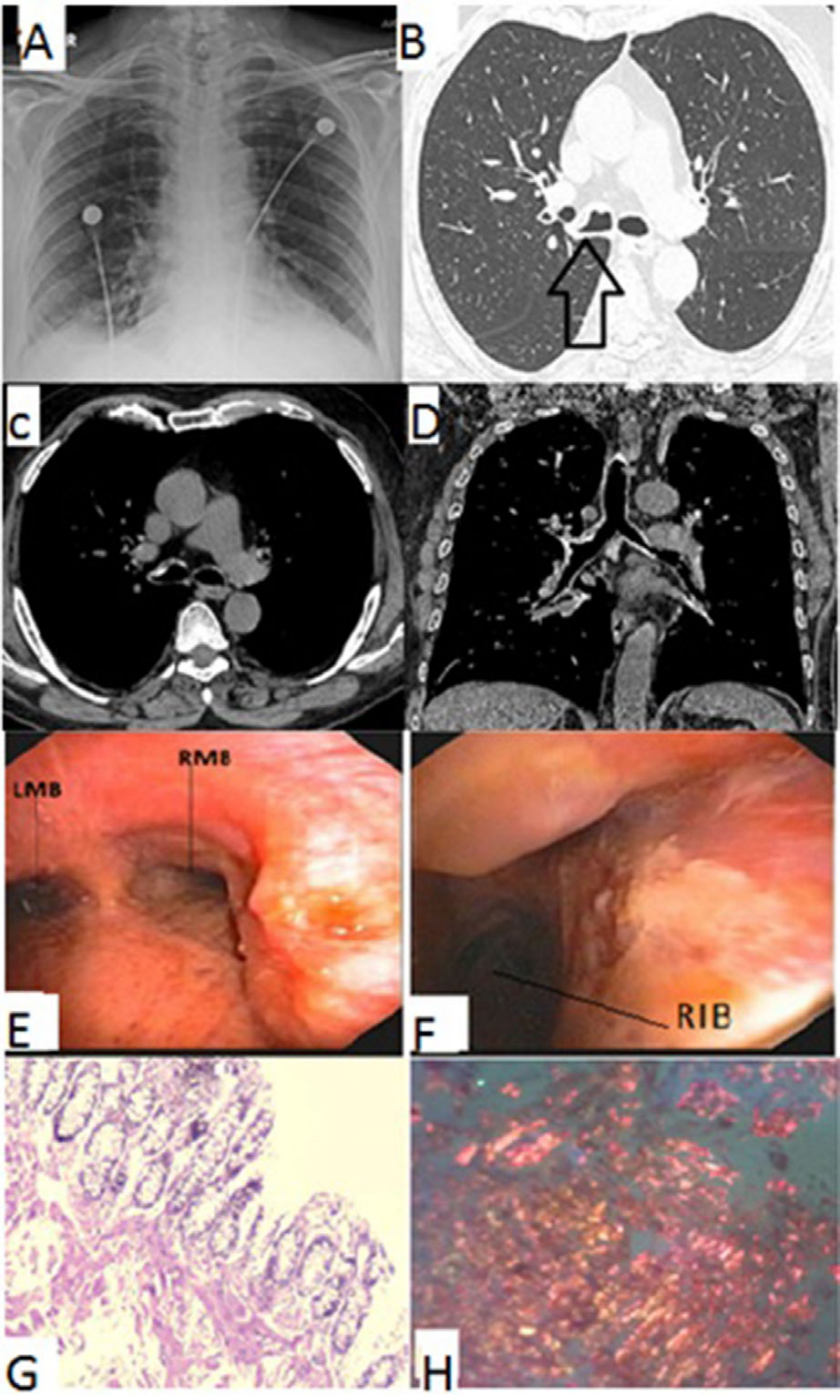
Findings of the patient in case 1. Radiology. (A) Chest X‐ray showed no parenchymal abnormality. (B) HRCT of the chest showing thickening in the wall of lower trachea and RMB. (C, D) Axial and coronal mediastinal sections of chest CT showing speckles of calcification were also seen. No other abnormality was seen. Bronchoscopy. (E) Endobronchial lesion with irregular mucosa in the lower part of trachea extending into the RMB. (F) Right main bronchus showing endobronchial lesion extending into the opening of right upper lobe. Histology. (G) Endobronchial biopsy showing pale pink homogeneous material suggestive of amyloid deposits. (H) Congo red staining demonstrating apple‐green birefringence confirming diagnosis of amyloidosis. CT, computed tomography; HRCT, high‐resolution CT; LMB, left main bronchus; RIB, right intermedius bronchus; RMB, right main bronchus.

A 70‐year‐old male, former smoker with known history of diabetes mellitus (DM), coronary artery disease (CAD), and poorly controlled chronic obstructive pulmonary disease (COPD) on regular treatment presented with progressive breathlessness and recurrent streaky haemoptysis. Initially, he was evaluated at other hospital where he underwent bronchoscopic biopsy from the endobronchial lesion which turned out to be inconclusive and later he was referred to our facility for further management. The patient was haemodynamically stable on admission. Chest X‐ray and high‐resolution computed tomography (HRCT) of the chest (Fig. 1) showed thickening of the wall of lower trachea and right main bronchus with no parenchymal abnormality. Because of persistent haemoptysis and airway thickening, video bronchoscopy was done which showed endoluminal lesion in the right lateral wall of lower trachea extending to the right upper lobe bronchus. Multiple biopsies of the lesion were taken (Fig. 1) and narrow band imaging showed sudden interruption of blood vessels. The diagnosis of amyloidosis was made from histopathology supported with special staining. Positron emission tomography‐computed tomography (PET‐CT) was done to rule out multi‐system involvement which showed no abnormal uptake of 18FDG (fluorodeoxyglucose) in the body. Argon plasma coagulation (APC) (ERBE, axial probe, power: 20 W, flow rate: 0.2 L/min) was applied to control the bleeding. Control bronchoscopy was performed at third and sixth months and the patient is presently doing well with no recurrence of symptoms.

### Case 2

A 60‐year‐old male, ex‐smoker, known case of COPD, got admitted with complaints of shortness of breath, cough with expectoration, and haemoptysis for three to four days. He had a history of tuberculosis treated adequately with anti‐tuberculous treatment twice in the past 20 and 10 years before the current admission. He also had a history of mild intermittent haemoptysis for last two years for which he underwent multiple admissions and bronchoscopy at other hospital with no conclusive diagnosis.

**Figure 2:**
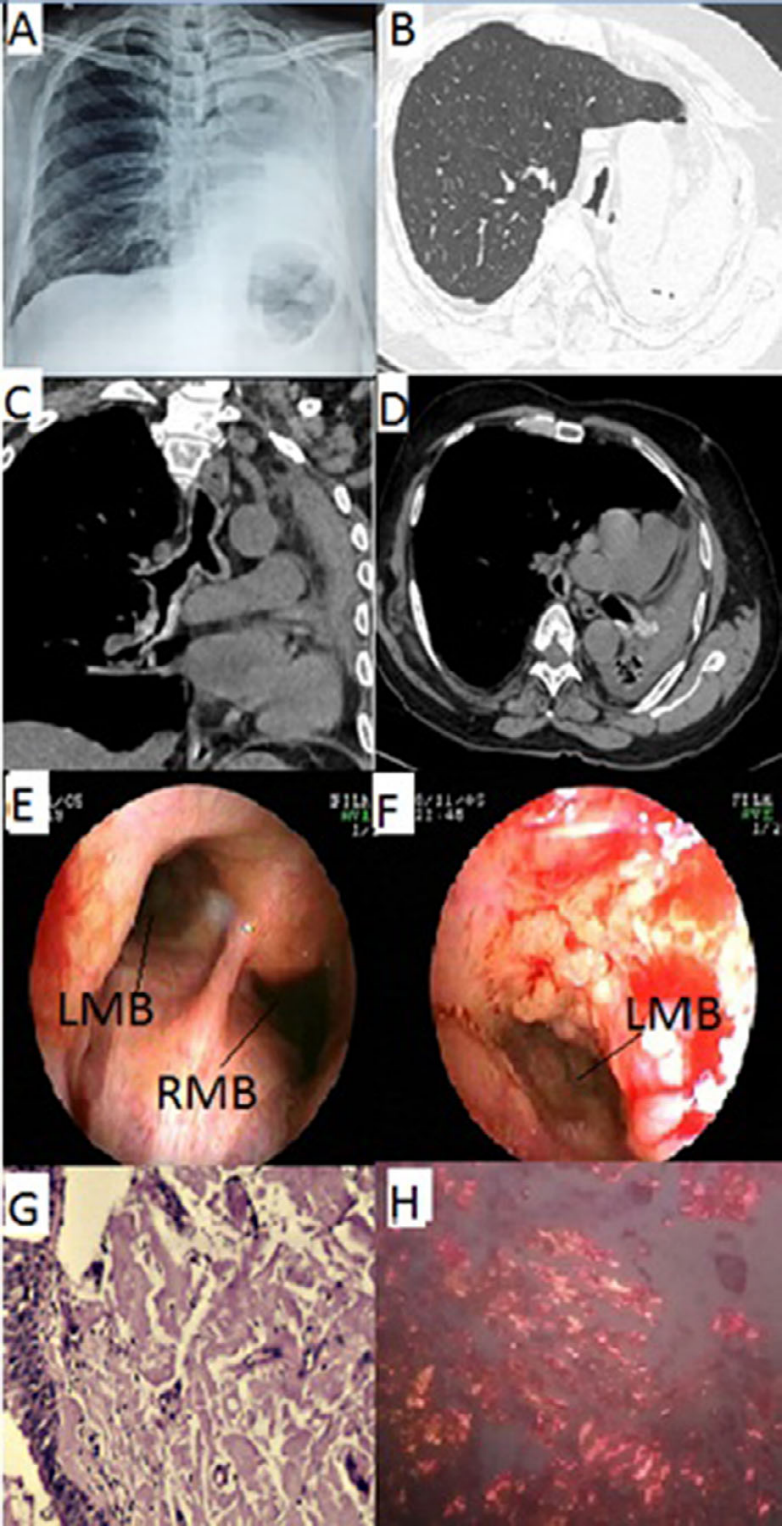
Findings of the patient in case 2. Radiology. (A) Chest X‐ray showing left lung collapse with ipsilateral mediastinal shift and hyperinflated right lung. (B) HRCT of the chest showing left lung collapse and right lung hyperinflation. (C, D) Axial and coronal mediastinal sections of chest CT showing speckles of calcification were also seen. No other abnormality was seen. Bronchoscopy. (E) Bronchoscopy showing deformed tracheal anatomy and narrowing of LMB and RMB with typical yellow coloured lesions bleeding on touch suggestive of amyloidosis. (F) Near‐complete occlusion of LMB with endobronchial lesion bleeding on touch. Histology. (G) Endobronchial biopsy showing pale pink homogeneous material suggestive of amyloid deposits. (H) Congo red staining demonstrating apple‐green birefringence confirming diagnosis of amyloidosis. CT, computed tomography; HRCT, high‐resolution CT; LMB, left main bronchus; RMB, right main bronchus.

The patient was admitted to our facility with haemoptysis for further management. Fibreoptic bronchoscopy showed distorted anatomy of entire tracheobronchial tree with typical yellow coloured lesions bleeding on touch suggestive of amyloidosis which was later histologically confirmed with Congo red staining (Fig. 2). He was treated with APC (ERBE, straight probe, power: 20 W, flow rate: 0.2 L/min) to control the bleeding. The patient was later discharged after haemostatic control and symptomatic relief. The patient was followed up after three months of discharge and control bronchoscopy showed no further haemoptysis from the lesion.

### Case 3

A 41‐year‐old male, non‐smoker got admitted with complaints of fever, cough with expectoration, and haemoptysis for three days. He was being treated for bronchial asthma for the last five years on inhaler therapy. He also had a history of recurrent mild intermittent haemoptysis for the last four months, which was managed conservatively without bronchoscopy and imaging.

The patient was admitted to our facility with haemoptysis for further management. Routine laboratory parameters, electrocardiogram, and 2D echocardiography were normal. Contrast‐enhanced CT (CECT) of the thorax was done which showed circumferential thickening and irregular lower tracheal and bronchial walls. The lung parenchyma and mediastinal structures were normal. Fibreoptic bronchoscopy showed distorted anatomy of the entire tracheobronchial tree with tumour‐like nodules bleeding on touch. Endobronchial biopsies with Congo red staining later confirmed amyloidosis. He was managed conservatively and was later discharged on symptomatic treatment. PET‐CT was done which showed no hypermetabolic focus in the body. Serum protein electrophoresis was done to rule out multiple myeloma which was normal. Control bronchoscopy was performed monthly for three months and then three monthly for a year post discharge. The patient is presently doing well and still continues to follow‐up six years after the first admission with occasional streaky haemoptysis with no further progression of other symptoms.

## Discussion

The lung presentation of amyloidosis can be described as tracheobronchial, nodular, and alveolar septal amyloidosis. The first two types are benign in nature, whereas alveolar type is often severe and progressive [1]. Isolated TBA is a rare entity, with only few case reports described in the literature. Although rare, these patients may present with common symptoms which can pose a diagnostic challenge; therefore, awareness of the possibility of the disease is essential for diagnosis and subsequent management. We described three cases of patients with undiagnosed recurrent haemoptysis which on further evaluation were found to have TBA.

A recent study suggests association between smoking and COPD with development of TBA. The precursor proteins responsible for amyloid synthesis are secreted by monocytes and macrophages, both cells are also increased in COPD pathogenesis [2]. The mean age at presentation was 50–60 years and males were more affected than females [3]. Two of our patients were elderly males and ex‐smokers who presented with recurrent haemoptysis and one patient was a relatively young and non‐smoker male.

Amyloidosis can produce submucosal plaques or tumour‐like masses, which may be localized or diffuse. The symptoms in patients with TBA arise due to local complications such as mucosal ulcerations, stenosis of the tracheobronchial tree, distal collapse, and recurrent pneumonia. Patients often present with common respiratory symptoms such as cough, shortness of breath, and haemoptysis.

Thoracic imaging in TBA may show circumferential airway wall thickening, tracheal stenosis, calcified extraluminal amyloid deposits, or mediastinal lymphadenopathy. Although there are case reports supporting the role of PET‐CT in diagnosis of TBA and pulmonary amyloidosis with subsequent follow‐up studies [4], there is no strong evidence to support the role for PET‐CT in diagnosis [5]. PET‐CT is often done to rule out any associated malignancies and other systemic conditions.

There was a diagnostic delay in our cases due to symptoms mimicking poorly controlled COPD for years. Recurrent haemoptysis warranted further investigation with imaging, bronchoscopy, and biopsy which confirmed TBA (as seen in the first and third cases). There have been few case reports where patients have been initially misdiagnosed as asthma and later found to have multiple endobronchial nodules diagnosed as amyloid [6]. Solitary endobronchial amyloidosis having a nodular appearance is present in 14% of patients and can mimic endobronchial tumour [7]. Thus, our cases highlight the importance of evaluation of poorly controlled obstructive disease with imaging and bronchoscopy to evaluate undiagnosed haemoptysis. It also showed that TBA can mimic endobronchial tumour and histopathology with special staining is essential for diagnosis [8].

Our second patient was initially managed as a case of post‐tuberculosis sequelae. Amyloidosis is considered as a long‐term complication of tuberculosis and should be considered as one of the differentials in evaluating a patient with long‐term post‐tuberculosis sequelae [9]. Due to progressive worsening of symptoms and haemoptysis, he was further evaluated to rule out malignancy and amyloidosis. Endobronchial finding was suggestive for amyloidosis and Congo red staining confirmed the diagnosis.

Amyloidosis is a multi‐systemic disease involving different organs including heart, kidney, and liver, which can range from asymptomatic to end organ failure. It is therefore important to rule out involvement of other organs to assess prognosis. Multiple myeloma must be included in the workup due to increased frequency of occurrence of AL amyloidosis with myeloma [5]. Our patients were evaluated for evidence of multi‐system involvement using imaging procedures and serum protein electrophoresis which did not show any evidence of other organ involvement and multiple myeloma.

The treatment of TBA is mostly conservative and symptomatic. Localized disease is managed by interventional procedures such as debridement with forceps, laser ablation, and APC which have shown to work well [10]. Debulking is usually required in central airway obstruction. In our first two cases, APC was used to achieve haemostasis with a good response. A large retrospective analysis of bronchoscopic interventions was carried out with good short‐term results. Long‐term outcomes depend on disease location and severity [8]. Proximal disease had more recurrences and high disease progression rates. Cryotherapy has been tried in TBA with good outcome [11]. Stents have also been used successfully in recurrent stenotic TBA following dilation [12]. The role of pharmacological drug therapy in TBA is unclear; however, it has been used in systemic diseases. The various drugs which have been tried are melphalan, dexamethasone, bortezomib, and haematopoietic stem cell transplantation with variable response [13].

The prognosis of TBA patients is variable with a mortality ranging from 30% to 50% after five to 10 years. One of our patients has been relatively asymptomatic for six years now. Long‐term follow‐up is therefore necessary for these patients to look for any progression of the disease.

TBA is an uncommon disease presenting with non‐specific symptoms. The diagnosis requires a high level of suspicion and has to be supported with relevant investigations. Monitoring the progression of the disease is helpful in predicting disease progression and need for further intervention.

### Disclosure Statement

Appropriate written informed consent was obtained for publication of this case series and accompanying images.
